# The current situation and strategy of Olympic education for primary and secondary school students based on Science- Technology- Engineering- Art- Mathematics education in the context of physical literacy

**DOI:** 10.3389/fpsyg.2022.910599

**Published:** 2022-07-28

**Authors:** Jia Li, Lei Yuan

**Affiliations:** ^1^School of Philosophy and Sociology, Jilin University, Changchun, China; ^2^College of Physical Education, Jilin University, Changchun, China

**Keywords:** physical literacy, STEAM education, Olympic education in primary and secondary schools, educational concept, the current situation of education

## Abstract

The purpose of this paper is the influence of Science- Technology- Engineering- Art- Mathematics (STEAM) education on the Olympic education of primary and middle school students. The research object is the Beijing Olympic model school. The frame structure and educational concept of STEAM education are studied, and a questionnaire survey on the current situation of Olympic education is conducted. Finally, improvement measures based on the survey results are provided combined with STEAM education, and the teaching effect is analyzed. The results show that after the improvement of the educational model, the student’s mastery of the Olympic knowledge has been greatly improved. The proportion of students who can master all knowledge increases from 5.78 to 8.45%, by 2.67%. The proportion of students with knowledge of most Olympic sports increases from 48.6 to 55.67%, by 7.07%. The proportion of students with little or no knowledge drops to 1.54%. Meanwhile, students are increasingly interested in Olympic events, especially after being inspired by the STEAM education model. The proportion of students who are very concerned about Olympic events has increased by 6.45%. The proportion of students who are more concerned about Olympic events has increased by 5.11%. Thus, the Olympic education work has achieved some results. Students gradually become interested in the Olympic events. They begin to actively pay attention to the Olympic events and learn the Olympic spirit. Then, their physical literacy is improved.

## Introduction

In terms of physical fitness, the expectations for sport-specific or competitive skills do not rank high. Parents want to exercise their children’s resilience, body shape, physical adaptability, physiology, and vision. The cultivation of physical literacy is an indispensable and important part of the all-around development of primary and secondary school students. However, they have very little time to do physical exercise due to the current academic pressure on primary and secondary school students. The beauty of speed and power in sports is an important material for artistic creation and the main source of artistic inspiration. At the cognitive level, parents generally recognize the importance of sports ability, but the related knowledge literacy needs to be improved. The school is not optimistic about the actual implementation of Olympic education. Science- Technology- Engineering- Art- Mathematics (STEAM) education has been widely recognized worldwide. Its role is undoubted, and its educational concept has also been tested in practice. It can cultivate comprehensive innovative talents. The original intention of establishing the International Olympic Committee is to play the educational role of the Olympic Games. The Olympic Games can inspire young people to work hard, and the Olympic spirit can be sublimated based on education (Zhang and Chen, 2021; Takahashi et al., 2022). Winter Olympic education is an important branch of Olympic education. The exploration of the role, influence, and value of Winter Olympic education in student development, Olympic movement development, and national sports development can promote the popularization of primary and secondary schools and the development of Winter Olympic cultural education (Weinmann et al., 2021). Applying the STEAM education concept in Olympic education in primary and secondary schools will help students better understand the Olympic spirit and help students stay in good shape.

Under the concept of STEAM education, the pedagogical education system aims to develop relevant people with the necessary competencies such as meta-discipline, design, and research skills (Qian et al., 2018; Rogoza et al., 2018; Chen, 2019). Anisimova et al. (2020) pointed out that one of the possible innovations to train teachers for STEAM education was to incorporate robotics into the training content of future teachers such as physics, mathematics, technology, and visual arts. Ribeiro et al. (2020) examined the impact of the Olympic Games by exploring the impact of the Olympic Games on the development of teachers’ skills, knowledge, and experience. Data were collected through an online questionnaire that included physical education teachers participating in the Transforma program. Structural equation modeling results showed that knowledge and skill development could measure teachers’ positive contributions to human development and their knowledge of Olympism teaching through experience. With the addition of the arts, student creativity was described as a key skill that must receive special attention. Aguilera and Ortiz-Revilla (2021) reviewed empirical educational interventions based on STEM and STEAM to determine their potential for developing students’ creativity. Attali and Yondre (2022) analyzed the positions and measures implemented by French educational actors regarding Olympism, focusing on professional texts for physical education and sports teachers. Finally, the content of a section of the project labeled “Olympic Class,” one of the main channels for promoting Olympic education in schools, is studied to complete this analysis. This study makes it possible to identify how teachers engage and grasp the concept of Olympic education, sometimes to the point of validating or altering its ideological basis (Wu and Song, 2019; Wu et al., 2020; Yuan and Wu, 2020).

In conclusion, although many scholars have different research perspectives on Olympic research, they all expound on the practical value of Olympic education. This paper provides new ideas for Olympic education in primary and secondary schools. This paper studies the current situation of Olympic education and STEAM education for primary and middle school students through literature research and a questionnaire survey. The innovation point is to give opinions and countermeasures based on the current situation of the Beijing Olympic model school combined with the STEAM education model. This is an outstanding contribution and can fill the research gap.

## Current situation of STEAM education and physical education

In recent years, emerging methods of applying and integrating science, technology, engineering, arts, and mathematics in education have emerged as a teaching alternative that provides a well-rounded and engaging education. STEAM education has received increasing attention over the past decade, mainly at the middle and high school levels. DeJarnette (2018) focused on the need for STEAM education in early childhood. Preschoolers had a natural inclination toward science due to their curiosity and creativity. This ethnographic study involved the professional development of 50 in-service preschool teachers in a high-needs area of an urban northeastern United States. Researchers provided hands-on professional development, consistent support, and extensive resources for STEAM curriculum implementation. They examined how incorporating their STEAM curriculum into the early childhood curriculum would affect teachers’ personality, self-efficacy, and implementation rates. Harris and De Bruin (2018) studied secondary schools in Australia, the United States, Canada, and Singapore. The research investigated how creativity was understood, valued, and embodied in secondary schools. The research focused on teachers’ and students’ understanding of creative and critical thinking. They examined how teachers fostered students’ creativity through teaching methods. Research results enhanced connectivity and interdisciplinarity between fields of study, impacting STEAM education, focused instruction, and educational change. Ozkan and Umdu (2021) explored the need for STEAM education to focus on developing students’ conceptual understanding of scientific content. The objective was to examine the effectiveness of STEAM education in developing 7th grade (13–14 years old) students’ conceptual understanding of strength and energy topics. An experimental embedded mixed methods design was used based primarily on quantitative data. A control group was used to determine the role of STEAM methods in students’ conceptual understanding. A STEAM approach was used for the study group, while the control group was taught according to the regular science curriculum. The test is carried out through the concept of force and energy. Research data were collected through semi-structured interviews with students in ten study groups.

Vasconcellos et al. (2020) examined the evidence for self-determination theory in the context of school sports. They applied a multilevel structural equation modeling approach to meta-analyze data from systematic reviews. The review identified 265 relevant studies. According to theory, autonomic motivation was positively associated with adaptive outcomes and negatively associated with maladaptive outcomes. Introverted regulation was moderately associated with adaptive and maladaptive outcomes. Jiménez-Barbero et al. (2020) assessed the link between sports and school violence and bullying. Relevant studies of quantitative and qualitative designs that met previously established eligibility criteria were identified by systematic searches in Medline, PsycINFO, SPORTDiscus, Web of Science, and Scopus. Varea et al. (2022) explored changes in physical activity during COVID-19 and the impact on pre-service teachers. Data collection was performed on 12 pre-service physical education teachers (4 women and 8 men) from Spain using semi-structured interviews. The results suggested that pre-service teachers had trouble reassembling sports in the age of COVID-19. This led to instability, fear, and insecurity. In addition, the reorganization of physical education will also lead to changes in teaching. The new mix of physical education also includes exposure to digital technology. This allows for specific openings and closures for realignment into mobile physical education. It is found that STEAM education positively impacts students’ conceptual understanding and reduces or changes the number of misunderstandings. The form of physical education changes with the times, and the combination of the two types of education is beneficial to the comprehensive quality education of future students.

## Materials and methods

### STEAM education in the context of physical literacy

**Figure 1:**
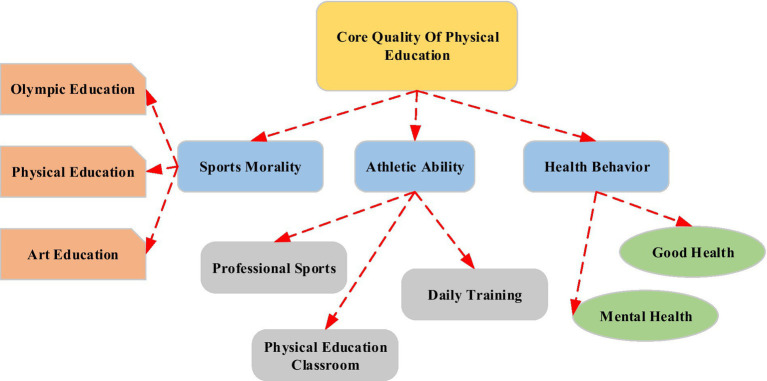
Core quality of physical education.

The development of children’s basic motor skills is mainly before the age of 10, which plays a crucial role in the development of their later athletic abilities and executive brain functions (Andreeva et al., 2020; Sattorov and Saidov, 2021). The cultivation of physical literacy of primary and secondary school students not only relies on parents and schools to pay attention to nutritional matching, and reasonably arrange children’s three meals a day and high-quality sleep. The Ministry of Education pointed out that students should have the necessary characters and key abilities that can adapt to the needs of lifelong development and social development. In the training of youth sports reserve talents in China, there is a long-term separation of sports and education in the competition system. If the youth sports competition system and the school competition system are not integrated at the national level, it will be detrimental to the cultivation of reserve talents. This pyramid-style sports competition system with multi-departmental cooperation and multi-subject participation must be jointly built (Ghorbani et al., 2020; Polet et al., 2020; Zhang et al., 2020). The sports development of primary and secondary school students is a huge network system. In specific practice, it is necessary to build a youth sports governance system and improve the modernization level of youth sports governance ability (Pérez-Ordás et al., 2021). Sports core literacy is mainly composed of three dimensions: sports skills, healthy behaviors, and sports ethics, and it reflects the unique qualities and key abilities of sports and health disciplines. Figure 1 shows the structure of the core quality of physical education.

Primary school is the initial stage of children’s education. The focus of education at this stage is not how much theoretical knowledge students have mastered, but how to help them develop good living and learning habits through scientific and reasonable education methods. Then students’ overall development of physical and mental health is cultivated. Physical education is the key content of primary education. At this stage, students’ quality can be enhanced, and manual skills and practical ability are promoted through sports. In addition, in the process of sports, it can increase students’ communication ability and teamwork ability. Therefore, students have a sense of collective honor and a strong competitive spirit. Then, a good outlook on life and values can be developed from an early age.

The Olympic Games are a model of the combination of sport and art. Art and sports have much in common, and they are both important forces to promote human progress and social development. The beauty of speed and power in sports is an indispensable material for artistic creation and the main source of artistic inspiration. The life experience of an artist is inseparable from sports. The passion and persistent exercise required in creation coincide with sportsmanship. The achievements in sports are an important carrier to enhance national cultural self-confidence. The Olympic Movement is the art of strength and beauty. It delights people’s bodies and minds with its unique cultural charm, and it also urges people to forge ahead with a strong humanistic spirit. By carrying forward the Olympic spirit to primary and secondary school students, the Chinese lofty ideals, firm beliefs, fighting spirit, and competitive sports art are integrated. Meanwhile, the world outlook, outlook on life, and values of primary and secondary school students are unified, essentially casting the spiritual pillar of the great rejuvenation of the Chinese nation. These struggles are significant for realizing the Chinese Dream and building a community with a shared future for mankind.

**Figure 2:**
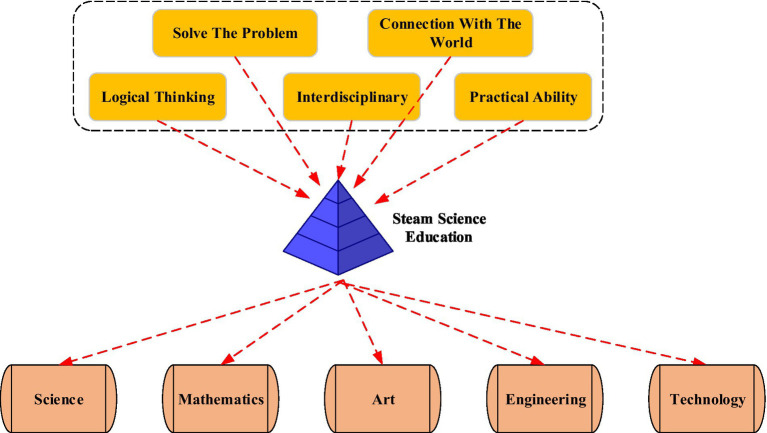
The structure of STEAM education.

STEAM is an acronym for the five disciplines of Science, Technology, Engineering, Art, and Mathematics. This term is often used in schools to enhance their competitiveness in technological development. It also appears in educational policy and curriculum planning, emphasizing that future students should develop interdisciplinary literacy and abilities. Its predecessor is Science- Technology- Engineering- Mathematics (STEM) education, that is, education in the fields of science, technology, engineering, and mathematics. The concept of art has been added to STEAM education. There is a connection between the five dimensions. Science and mathematics are the foundation. Art and mathematics are connected. Engineering is the goal. Technology is the means and process to achieve the goal. It is an interdisciplinary integration project learning oriented to real problems. In essence, it guides students into the real STEAM creation process and allows students to enter the learning process with a new attitude. STEAM education focuses on thinking skills and learning methods. In the STEAM project, students learn methods such as logical reasoning and hypothesis testing. Then, they will explore the subject knowledge in-depth and become better problem solvers. The structure of STEAM education is shown in Figure 2.

**Figure 3:**
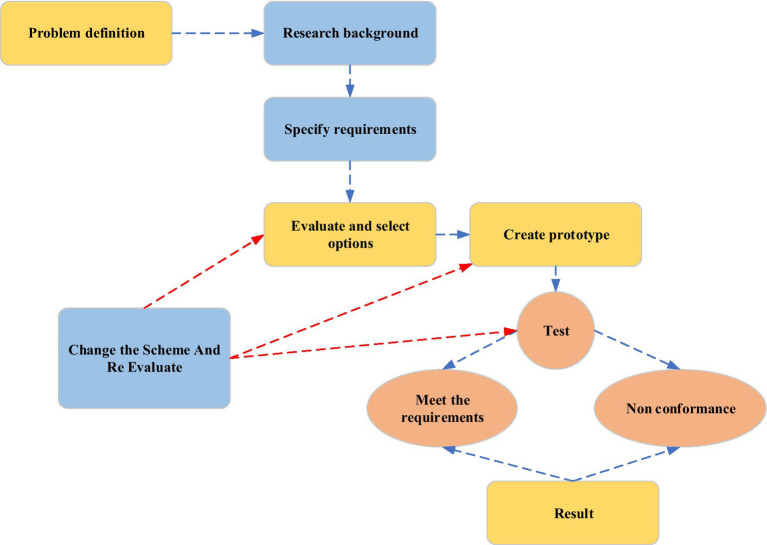
STEAM education teaching design process.

From Figure 2, STEAM has the characteristics of interdisciplinary, artistic sense cultivation, situational, cooperation, and the formation of design concepts. It takes the unique learning process as the core, emphasizes that students solve real-world problems through interdisciplinary knowledge, and encourages students to actively explore. The Olympic spirit is a “faster, stronger, higher” self-challenge spirit. Meanwhile, it is also the spirit of fairness, justice, equality, and freedom of sports competition. The spirit of self-challenge and fair competition contained in the Olympics constitutes the cornerstone of contemporary human self-improvement and social interaction. The purpose of Olympic education is naturally carried out around the Olympic spirit. It is largely consistent with the goals of STEAM education and teaching. The instructional design needs to reflect humanity. Students feel the truth, kindness, and beauty in the activity. In the process, the enthusiasm of students is stimulated to improve humanistic quality. Therefore, it is a good attempt to combine the two. The commonly used instructional design process is shown in Figure 3.

There are usually three teaching modes for STEAM courses: problem-based teaching, design-based teaching, and project-based teaching. Problem-based teaching extends the real problems in the environment to teaching activities. It stimulates students to practice science, engineering, technology, and mathematics in the process of scientific inquiry. When students encounter difficulties, teachers are not eager to guide but let students obtain solutions through their divergent thinking. Then, students can quickly apply the results of scientific inquiry to practice. The core of design-based teaching is the project-based inquiry teaching method developed for the learning of junior high school students. Students learn scientific knowledge through the form of design, to exercise their problem-solving ability. Project-based teaching is a combination of the first two. It uses project research as an entry point for scientific inquiry or engineering design or both.

### Investigation and analysis of the status quo of Olympic education in primary and secondary schools

The current situation of Olympic education was investigated in the 2022 Beijing Winter Olympics education model schools. According to the needs, a questionnaire on the education situation of the Olympic education model schools in Beijing primary and secondary schools was developed. The questionnaire survey was conducted on a total of 90 schools including primary schools, junior high schools, and senior high school model schools. The content of the questionnaire included the basic information of the school, the construction status of the Olympic education model school, existing problems, improvement measures, and suggestions. Experts in sports sociology and the head of the secretariat of the Olympic Education Working Group Office in primary and secondary schools were invited to form an expert team to test the reliability and validity of the questionnaire after it was completed. A total of 90 questionnaires were distributed and 90 were recovered, with a recovery rate of 100%.

The study used “test–retest reliability” to test the reliability and stability of the questionnaire. Half a month before the official questionnaire was issued, 15 model schools were randomly selected. Each school was issued a questionnaire and personal information was registered. A second survey was conducted on the principals of these 15 schools half a month later. The results were obtained: the correlation coefficient *R* = 0.851, and the tested significance level *p* < 0.01. It showed that the questionnaire has high reliability and stability. Then, a formal questionnaire was distributed. The data were analyzed using comparison, induction, analysis, and summary, combined with professional knowledge of sports, education, and art. In addition, SPSS20.0 was used. The reliability and validity meet the requirements. Finally, suggestions for improvement were given.

### Strategies for Olympic education in primary and secondary schools based on STEAM education

The importance and diversity of STEAM education concepts are shown above. Increasing the perspective of thinking for primary and secondary school students in the learning process is conducive to the comprehensive training of students’ abilities in different aspects, such as cognitive ability. Instrumental training can also improve students’ verbal and non-verbal skills. Given the investigation on the current situation of Olympic education in Chinese schools, some feasible measures to change the current situation are given.

The first is the integration of home and school, teachers and parents need to change their outdated way of thinking. Physical education teachers and parents of students should change the traditional educational concept, and encourage students to actively participate in their favorite sports activities. Then, students will re-establish sports goals and master the scientific method of self-exercise. Secondly, as the main body of Olympic education and teaching, physical education teachers first need to improve their comprehensive ability, to bring more knowledge to students. The professional quality of physical education teachers needs to be continuously improved, including cultural quality, psychological quality, and moral quality. The professional quality and comprehensive quality of physical education teachers have an obvious influence on students. Mr. Tao Xingzhi said: “In common life, teachers must strive to improve. Good students are always happy to compete with teachers in terms of knowledge and self-cultivation.” Therefore, physical education teachers should constantly improve themselves and improve their professional quality. They are to lead by example and be role models.

In addition, the school teaching model needs to combine Olympic knowledge with real life. School teaching should move from school to life, pay attention to problem orientation, and focus on the situation creation of Olympic sports and competition. Students need to learn and master structured Olympic sports knowledge and skills. School teaching should also enable students to use structured knowledge and skills to solve practical problems in real activities or competition situations. In physical education, students face various complex situations. They use their bodies and intelligence to challenge themselves, defeat opponents, feel self-confidence, success, and other complex physical and mental experiences. It is also a process to promote students’ physical and mental health and develop core literacy. Physical education teachers should learn to explore independently and cultivate students’ habits of thinking. Physical education teachers must first ensure that students explore and learn independently under a suitable exercise load. Then, they cultivate students’ ability to explore and learn, to truly realize the transformation from teaching-centered to learning-centered. Teachers evaluate whether the core literacy of students’ Olympic education has been formed from the three dimensions of sports skills, healthy behavior, and sports morality through students’ behavioral performance. Physical education teachers should emphasize moral education in teaching. Moral education should be run throughout the Olympic teaching classroom and carried out by combining the content of teaching materials and teaching activities in a targeted manner. For example, physical education teachers carry out collective collectivism education for students in-game competitions. Then, students can establish a good sense of caring for others, learn to live in harmony with others, and tolerate others. In a word, through the study of Olympic education, students can master and apply Olympic knowledge and skills, gain sports fun, and experience success. It also meets the needs of students to participate in Olympic sports.

Due to many students in the model schools, it was difficult to issue questionnaires. Therefore, after the above strategies were improved and implemented in these normal schools for one semester, a questionnaire survey was conducted on teachers of these model schools again. The effect of STEAM education in Olympic education was analyzed.

## Results and discussion

### The survey results of Beijing Olympic education model schools in primary and secondary schools

**Figure 4:**
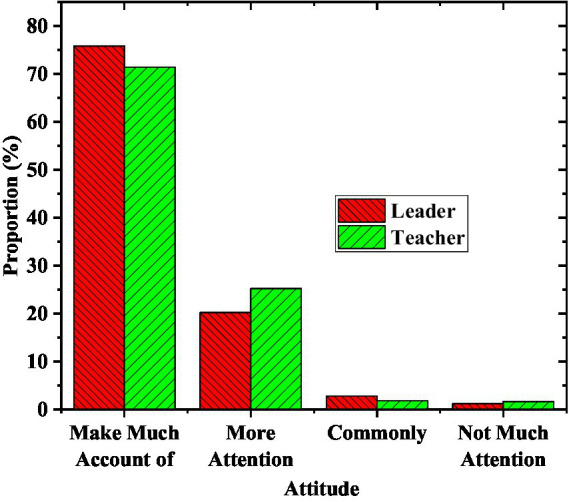
The attitude of school officials in the demonstration area toward Olympic education.

School officials fundamentally determine the process of Olympic education. The survey on leadership attitudes is displayed in Figure 4.

**Figure 5:**
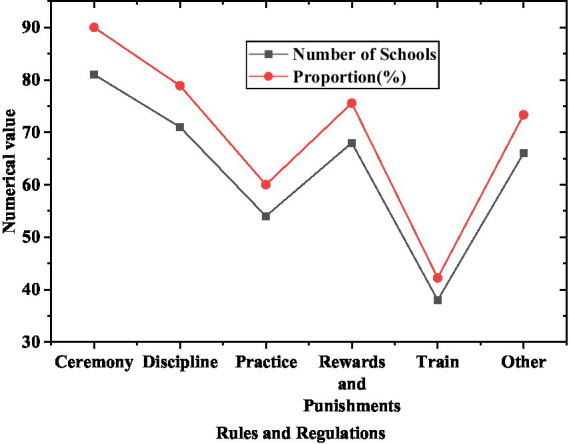
Model school rules and regulations construction.

From the comparison in Figure 4, 75.8% of school officials and 71.4% of teachers attach great importance to Olympic education. The proportion of people who maintain a general attitude and a low attitude toward Olympic education is relatively small. Most officials attach great importance to the school’s Olympic education work. Attitude support is one aspect, and whether the specific work has been implemented is the key to the advancement of education work. The content of Olympic education rules and regulations of the model schools participating in the survey is revealed in Figure 5.

From Figure 5, it can be found that 90% of the model schools have rules and regulations, including daily teaching content, and 78.99% of schools have civilized etiquette. There are 71 schools in total. More than half of the schools have safety discipline. It shows that most of the rules and regulations of Olympic education model schools are mainly based on the content of daily teaching activities, supplemented by other civilized etiquette and teacher training.

**Figure 6:**
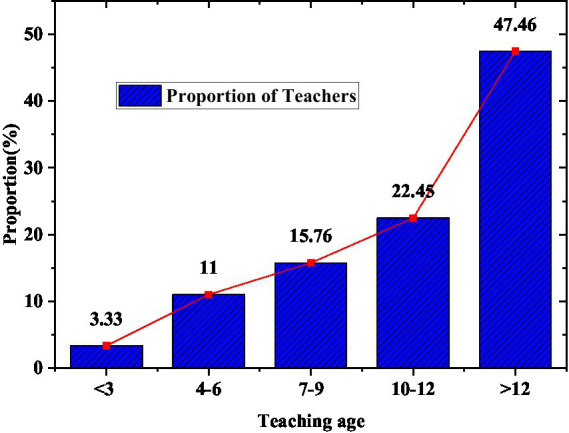
Teacher’s teaching age.

**Figure 7:**
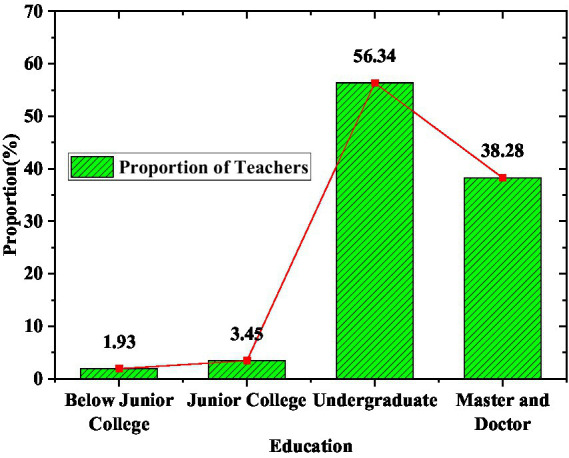
Composition of teachers’ academic qualifications.

As the body of education work, teachers are responsible for curriculum teaching and organizing educational activities in Olympic education work. They play an important role in popularizing Olympic education in schools. The composition of teachers in the survey subjects is shown in Figures 6, 7.

Figures 6, 7 indicate that nearly half of the teachers in the teaching team have more than 12 years of teaching experience. The proportion of teachers with a bachelor’s degree or above in the composition of teachers’ educational qualifications reached 94.62%. This result shows that most of the teachers in the surveyed model schools have good professional knowledge. Most of them are highly educated. They have good learning abilities and can better participate in the Olympic education work.

**Figure 8:**
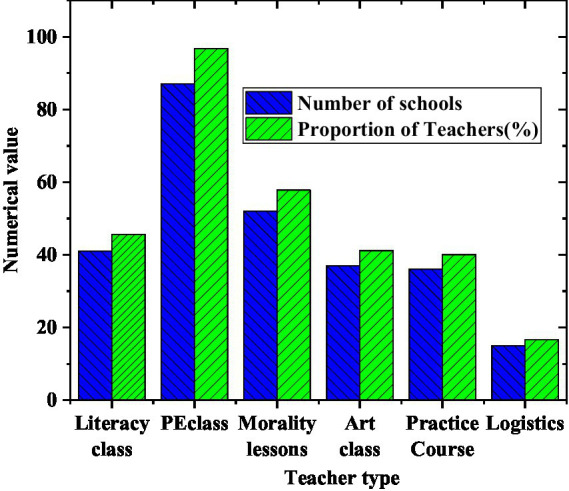
Teacher participation.

The composition of teachers involved in the implementation of Olympic education is shown in Figure 8.

Figure 8 shows that culture teachers from 41 of the 90 colleges and universities participated in Olympic education, accounting for 45.56%. The proportion of physical education teachers participating in Olympic education teaching accounted for 96.67%. It indicates that in the arrangement of most model schools, physical education teachers have become the main force of Olympic education, and physical education has become the main battlefield of teaching.

**Figure 9:**
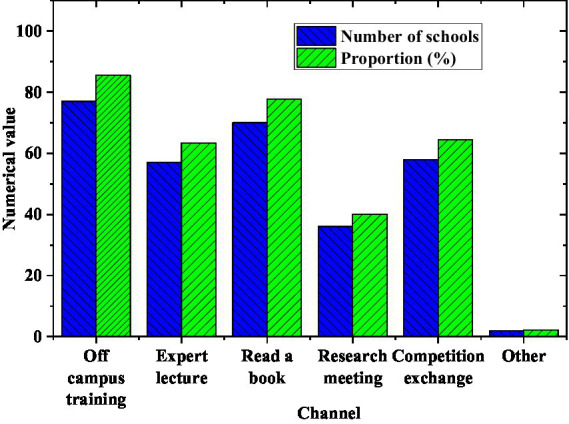
The way teachers improve their professional level.

Figure 9 displays the results of the survey on how teachers improve their professional level.

Figure 9 indicates that the way most teachers choose to improve their comprehensive business level is to go out for training, and the proportion reaches 85.56%. Secondly, 57 schools choose to hire well-known experts to do related training in schools, accounting for 63.33%.

**Figure 10:**
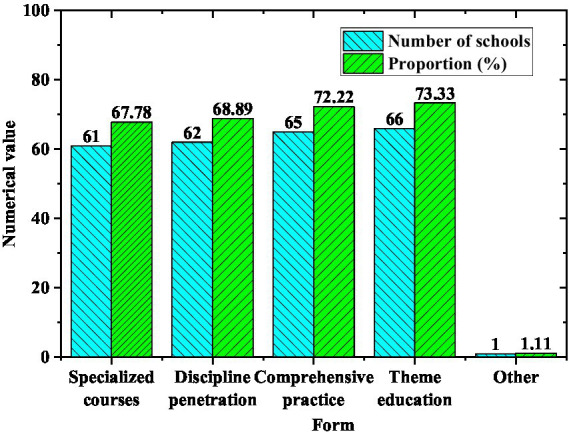
The teaching form composition of Olympic education in model schools.

The teaching form of Olympic education in each model school is shown in Figure 10.

From Figure 10, 61 schools provide students with Olympic education through the education of special courses, accounting for 67.78%. 68.89% of schools adopt the education infiltration of other disciplines. 72.22% of schools adopt the organization of comprehensive practice activities to popularize Olympic knowledge. Therefore, most of the schools have diversified teaching forms and cover a relatively comprehensive range of teaching.

### Teaching effectiveness of Olympic education

**Figure 11:**
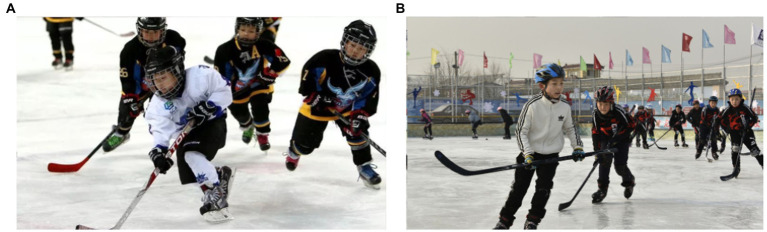
The teaching situation of the Olympic sports in the school [**(A)** and **(B)** represent the real pictures of different links of the Olympic sports]. Reproduced with permission from Beijing Yanqing District Education Commission, available at https://m.sohu.com/a/315362623_161623.

Figure 11 shows the implementation of the Olympic Games from the Internet in schools.

**Figure 12:**
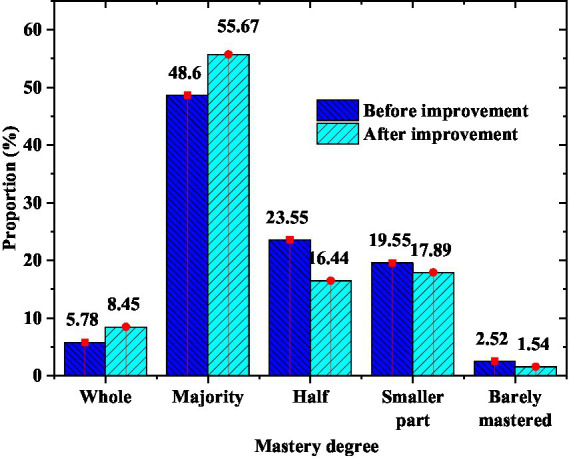
Students’ mastery of knowledge.

From Figure 11, the Olympic education model schools have various forms of Olympic events, and students have a high degree of participation and interest. Especially in comprehensive practice activities, students feel the Olympic spirit through the experience of practical projects. After improving the teaching model of the model school in combination with the STEAM education model, the student’s mastery of Olympic knowledge is shown in Figure 12.

Figure 12 reveals that the students’ knowledge mastery has been greatly improved after the adjustment. The proportion of students who can master all knowledge increased from 5.78 to 8.45%, by 2.67%. The proportion of students with knowledge of most Olympic sports increased from 48.6 to 55.67%, by 7.07%. The proportion of almost no knowledge dropped to 1.54%. This result shows that the teaching effect of Olympic education combined with STEAM education has been promoted.

**Figure 13:**
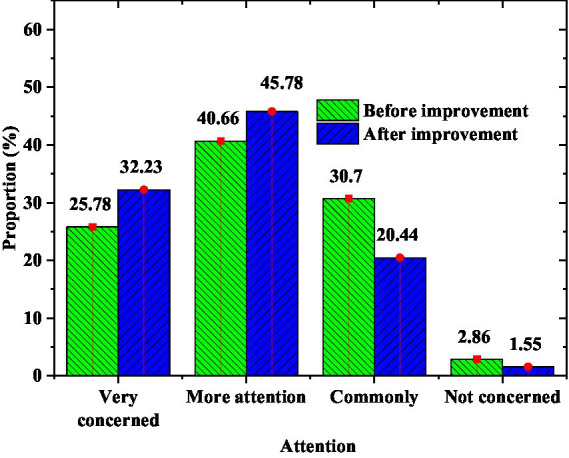
Students’ attention to Olympic events.

Changes in students’ attention to Olympic events are shown in Figure 13.

Figure 13 shows that students pay more and more attention to Olympic events after Olympic education. Especially after being inspired by the STEAM education model, the proportion of students who are very concerned about Olympic events has increased by 6.45%, and the proportion of students who are more concerned about Olympic events has increased by 5.11%. The percentage of students who did not care about Olympic events at all fell by 1.31 percent.

STEAM is a popular teaching method to improve students’ creativity, problem-solving skills, and interest in STEM fields, but the definition and purpose of STEAM education remain ubiquitous, especially in connection with physical education (Perignat and Katz-Buonincontro, 2019). Over time, STEAM research does not have an established and robust line, although trends in this regard can be observed to focus on the scientific branch of education (Marín-Marín et al., 2021). This paper combines the STEAM education concept with Olympic education to improve students’ concepts and the educational effect of sports compared with the research in related fields. This indicates the importance of Olympic education. The school’s Olympic education work has achieved some results. Students gradually become interested in the Olympic events, actively pay attention to the Olympic events, and learn the Olympic spirit.

## Conclusion

Whether in off-campus training institutions or public schools, STEAM is an acronym for the five disciplines of Science, Technology, Engineering, Art, and Mathematics. The development of STEAM education in China is in its infancy. Chinese students urgently need to be liberated from the strong pressure of exam-oriented education. It requires the guidance of the government, the cooperation of schools, and the support of social forces. As a synthesis of art and sports, the Olympic Movement coincides with the concept of STEAM education. Primary and secondary school students need this kind of educational method. The method has achieved results to change the existing Olympic education situation, which is also conducive to the cultivation of students’ physical literacy. Through the research on STEAM education and the investigation of the current situation of Olympic education, there are still many problems in the current Olympic education. However, the situation is improved through the STEAM education concept. Besides, there are still some deficiencies in the research. STEAM education has high requirements on teachers, and now domestic normal schools have not yet opened STEAM majors, which makes many teachers lack professional knowledge. Moreover, the schools mainly focus on taking exams, and the educational philosophy of most schools has not undergone a fundamental change. In future research, parties need to work together to truly change the current situation of Olympic education. Only some Olympic education model schools in Beijing are surveyed, and the amount of data is small, and much data support is needed in future work.

## Data availability statement

The raw data supporting the conclusions of this article will be made available by the authors, without undue reservation.

## Ethics statement

The studies involving human participants were reviewed and approved by Jilin University Ethics Committee. The patients/participants provided their written informed consent to participate in this study. Written informed consent was obtained from the individual(s) for the publication of any potentially identifiable images or data included in this article.

## Author contributions

All authors listed have made a substantial, direct, and intellectual contribution to the work and approved it for publication.

## Conflict of interest

The authors declare that the research was conducted in the absence of any commercial or financial relationships that could be construed as a potential conflict of interest.

## Publisher’s note

All claims expressed in this article are solely those of the authors and do not necessarily represent those of their affiliated organizations, or those of the publisher, the editors and the reviewers. Any product that may be evaluated in this article, or claim that may be made by its manufacturer, is not guaranteed or endorsed by the publisher.
